# Sociodemographic Characteristics, Dietary Practices, and Nutritional Status of Adults with Hypertension in a Semi-Rural Community in the Eastern Region of Ghana

**DOI:** 10.1155/2018/2815193

**Published:** 2018-07-18

**Authors:** Esi K. Colecraft, Matilda Asante, Aaron K. Christian, Seth Adu-Afarwuah

**Affiliations:** ^1^Department of Nutrition and Food Science, University of Ghana, P.O. Box LG 134, Legon, Accra, Ghana; ^2^Department of Dietetics, School of Biomedical and Allied Health Sciences, College of Health Sciences, University of Ghana, Accra, Ghana; ^3^Regional Institute for Population Studies, University of Ghana, Legon, Ghana

## Abstract

**Introduction:**

Hypertension is a major contributor to the global disease burden and mortality. Evidence suggests increasing hypertension prevalence in Ghana but there is limited public awareness and information on the characteristics of those with the disease.

**Objective:**

To describe the baseline characteristics of adults with hypertension who were randomized to receive either hypertension related nutrition education plus hospital-based standard of care or only the standard of care (control group) in Asesewa, a semi-rural community in the Eastern Region of Ghana. Only baseline data were used in the present analysis.

**Methods:**

A cross-sectional baseline survey was completed for 63 adults with confirmed hypertension diagnosis. Data on sociodemographic characteristics and diet were obtained through interviews and participants' body mass index (BMI) was computed. Pearson chi-square statistic was used to assess differences between those with both elevated diastolic blood pressure (DBP) and systolic blood pressure (SBP) and those with only elevated SBP.

**Results:**

Mean ± SD age of participants was 54.5±13.8 years. Approximately 71% of participants had both elevated SBP (>140 mmHg) and DBP (>90 mmHg) while the remaining 29% had only elevated SBP. The median number of times the food groups beneficial to hypertension management were consumed in the preceding week to the interview was 1 for green leafy vegetables, 1 for dairy products, 2 for fruits, and 4 for legumes. The median number of times for consuming harmful food groups was 3 for salted fish and 7 times for fats and oils. Signifcantly more participants in the age group above 50 than the younger participants had elevated SBP (83.3 vs. 16.7:P=0.027) and those with BMI equal to or greater than 25 were more likely to have both elevated SBP and DBP (P=0.047).

**Conclusions:**

Findings from this study have implications for the prevention and management of hypertension in this semi-rural population.

## 1. Introduction

The decision to render the phrase “*Control your blood pressure*” as the theme for 2013 World Health Day signified increasing recognition that elevated blood pressure (EBP) or hypertension is an important global public health concern [1]. Similarly, noncommunicable diseases were highlighted in the 2012 World Health Statistics report in which hypertension was estimated to contribute to more than one-half of the deaths from stroke and about 45% of deaths from heart disease [2]. The report also showed that while hypertension prevalence declined in most industrialized countries between 1980 and 2008, the opposite occurred in most African countries. With an estimated hypertension prevalence of nearly 37% in 2008, the African region had the highest prevalence of EBP. In Ghana, early reports suggested hypertension prevalence of less than 10% and less than 20% in rural and urban populations, respectively [3]. However, a 2010 systematic review revealed that, between 1970 and 2007, hypertension prevalence was between 19% and 48% [4]. While most studies generally reported lower hypertension prevalence for rural compared to urban localities, an assessment of hypertension prevalence in four rural communities in the Ga District of the Greater Accra Region reported rates of 25% and 37% among traders and farmers, respectively, leading to the authors' conclusion that hypertension is not only an urban concern in Ghana. Most Ghanaians with EBP in past studies were unaware that they had the condition [5].

Despite the increasing burden of hypertension in Ghana, there is limited context specific information on the sociodemographic and lifestyle risk factors of those affected. Identifying such risk factors is important for intervention planning, especially in a low resource context where lifestyle related preventive actions may be important in averting progression of mildly hypertensive or prehypertensive individuals to a more serious disease state requiring costly medical intervention.

Hypertension related deaths in low- and middle-income settings could potentially be reduced substantially by applying lessons learnt in high-income countries. One diet-based intervention that has been efficacious in reducing blood pressure among those with hypertension as well as preventing hypertension is the Dietary Approaches to Stop Hypertension (DASH) diet pattern developed in the United States and successfully adapted elsewhere including South Africa [6, 7]. The DASH diet promotes a dietary pattern that supplies nutrients shown to be “beneficial” (specifically, potassium, magnesium, and calcium) in the prevention and management of hypertension while minimizing “less beneficial” or “potentially harmful” nutrients (e.g., sodium and saturated fat and cholesterol) with respect to their effects on blood pressure [8]. The DASH dietary pattern emphasizes reducing sodium (salt) intake, increased consumption of fruits, vegetables, and low-fat dairy products and moderate consumption of whole grains, nuts, fish, and poultry. The DASH diet is based on evidence indicating that an increase in the consumption of fruits and vegetables has consistently been shown to be associated with lowering risk of hypertension [9]. Recommendations concerning reducing the consumption of meat and poultry are also consistent with the increasing number of studies indicating that their increased intake increases hypertension risk [10]. Epidemiological studies on the relationship between the consumption of whole grains and hypertension are far from being consistent. While some studies have found a lower risk of coronary heart disease others do not [11, 12]. Reduction in salt intake is strongly recommended as one of the top priority actions to tackle noncommunicable diseases and reduction of deaths from hypertension [13].

In 2015, a randomized controlled trial to test the impact of a nutrition education intervention based on the DASH diet principles on blood pressure was implemented among adults with blood pressure in Asesewa, a semi-rural township in the Eastern Region of Ghana. Baseline data collection on the study population (to facilitate pre- and postintervention comparisons of outcomes of interest) permitted the current analysis, which was aimed at describing the sociodemographic characteristics, dietary habits, and nutritional status of adults with hypertension enrolled in the nutrition education intervention study.

## 2. Methods

### 2.1. Study Area

The survey was carried out in Asesewa in the Upper Manya Krobo District in the Eastern Region of Ghana. Asesewa has a youthful population (50.9%) representing a broad-based pyramid with an elderly population of about 6%. Most people in the area (73.0%) are employed in the agricultural sector [14].

### 2.2. Study Design and Participants

This was a cross-sectional, baseline study of adults enrolled in the DASH-based nutrition education intervention study between February and July 2015. Potential participants were adults (>25 years old) who responded to announcements at places such as the main market, selected local churches, and the Asesewa Government Hospital at Asesewa for free blood pressure screening as well as adults attending outpatient services at the Asesewa Hospital, who had elevated BP.

Those screened with elevated blood pressure (systolic blood pressure (SBP) ≥ 140 mmHg and/or diastolic blood pressure (DBP) ≥ 90 mmHg) and those with EBP who were identified from the hospital OPD were asked to complete two consecutive follow-up measurements at the Asesewa Government Hospital to confirm the diagnosis. Persons with a confirmed diagnosis who agreed and signed the informed consent document after the study procedures had been explained to them were recruited into the study. Ethical approval for the study was granted by the Institutional Review Board of the University of Ghana Noguchi Memorial Institute for Medical Research. All participants signed or thumb-printed (if illiterate) a written informed document before being recruited.

### 2.3. Study Procedures

Blood pressure (BP) screening was done by trained project personnel using the digital Omron Blood Pressure Monitors (HEM-759-E, Omron Corporation, Kyoto, Japan), while the BP measurements to confirm hypertension diagnosis were completed by the nursing staff at the Asesewa Hospital. At enrolment, a pretested questionnaire was used to collect information on background sociodemographic characteristics, personal and family history of chronic diseases, and history of alcohol consumption and cigarette smoking. A 7-day food frequency questionnaire (FFQ) was used to obtain information about the number of times different food groups were consumed in the past 7 days. The FFQ was based on foods typically consumed in the locality and grouped in such a way to enable assessment of whether study participants' intake is in accordance with the DASH dietary pattern. Salted fish was added as a separate food group because it is a regular ingredient in Ghanaian cuisine which contributes significantly to salt intake with potential implications for hypertension. Additionally, participants' weights were taken to the nearest one kilogram with an Omron Body Composition Analyzer and Scale (Omron Healthcare, Inc., Vernon Hills, IL, USA). Participants' heights were taken with a Stadiometer to the nearest 0.1 centimetre. Anthropometric measurements were taken in duplicate and the averages computed.

### 2.4. Data Analysis

We performed statistical analysis by using Stata package 12 (STATA Corporation, Texas, USA). Body mass index (BMI) was computed from weight and height measurements and participants were categorized as underweight (BMI: < 18.5), normal weight (BMI: 18.5–24.9), overweight (BMI: 25.0–29.9), and obese (BMI: > 30.0). Descriptive statistics, mean ± SD for continuous variables and frequencies (%) for categorical variables, were used to summarize the data. The data on consumption of different food groups in the past week were skewed with some of the food groups having outliers; therefore median values were also computed. Bivariate analyses using Pearson Chi-Square Statistic were used to assess whether there were significant differences in sociodemographic characteristics, dietary intakes, and nutritional status between study participants with both elevated SBP and DBP and those with isolated only SBP.

## 3. Results

*Sample*. A total of 1165 adults were screened for hypertension and about 24% (n=282) had elevated systolic blood pressure. Only sixty-three adults with confirmed elevated systolic and diastolic blood pressure completed the baseline survey. Of these approximately 29% (16) had systolic hypertension only, while the remainder had both elevated systolic and diastolic blood pressure. Mean ± SD systolic and diastolic blood pressure of study participants were 151.9 ± 7.4 and 92.5 ± 7.7, respectively.

*Sociodemographic Characteristics of Respondents.* About 56% of the respondents were female, with a mean ± SD age of 54.5±13.8 years (Table 1). The majority were of Krobo ethnicity (76.2%) and Christian faith (90.5%). Approximately one-fifth of respondents did not have any formal education and only 17% had the tertiary level education. At the time of the survey, only a few (3.2%) of the participants were unemployed. The most common occupation was petty trading (38.1%) and about 22% of respondents had salaried occupations. Approximately 56% of the participants were married or lived with a partner (cohabiting). The mean household size was 5.1 (SD 2) persons.

Reported monthly household income was less than 200 Ghana Cedis (1 Ghana Cedi is approximately 0.23 US dollars) for the majority of participants and only 13% of households had incomes higher than 600 Ghana Cedis per month.

### 3.1. Nutritional Status of the Study Respondents and Family History of Chronic Conditions

The mean ± SD BMI of the respondents was 27.1 ± 5.3 with 38.1% and 25.4% of the respondents being overweight and obese, respectively. About one-fifth of study participants reported having at least one family member being overweight or obese. A family history of hypertension (55%) was the most common followed by stroke (23.8%). About 16% of the respondents reported a family history of diabetes and 8% had relatives that have suffered a heart attack.

### 3.2. Dietary Intakes of Respondents in the Preceding Week

**Figure 1:**
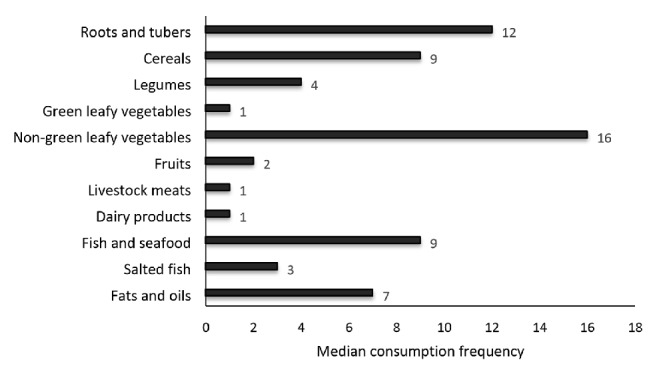
Respondents' median frequency of consuming foods from different food groups in the past seven days.

Non-green leafy vegetables, roots and tubers, and cereals were the most frequently consumed foods by the respondents in the past week (Figure 1). The median frequencies of consumption of these foods were 16, 12, and 9, respectively. The median frequencies of consumption of legumes, green leafy vegetables, and fruits were 4, 1, and 2, respectively, in the past week.

Fish and seafood were the most consumed animal source of foods. Salted fish and fats/oils were consumed a median of 3 and 7 times, respectively, in the past week.

### 3.3. Comparison of Sociodemographic Characteristics, Family History of Chronic Disease, and Nutritional Status between Participants with Both High Systolic and Diastolic BP and Those with Only Isolated Systolic BP

Summary of comparisons of sociodemographic factors, family history of chronic diseases, and nutritional status between participants with both elevated SBD and DBP and those with only isolated elevated SBP using Pearson Chi-Square Statistic is shown in Table 2. A significantly greater proportion of respondents who were below 50 years had both elevated systolic and diastolic blood pressure (46.7% versus 16.7%;* p*=0.027) and a greater proportion of obese/overweight individuals had both elevated systolic and diastolic pressure compared to individuals within the normal body size (71.1% versus 44.4 %;* p*=0.047) (Table 2).

Other sociodemographic factors such as family history of hypertension, income, and dietary intakes showed no significant difference among the various categories.

## 4. Discussion

This study contributes to our current knowledge of the characteristics and potential predictors of hypertension among adults living in semi-rural communities in Ghana. It is important to note that less than 50% of those screened with elevated BP returned for the necessary confirmatory tests to determine their hypertension status. This apathy to follow-up on screening results may reflect lack of awareness about hypertension and its consequences which have been noted among socioeconomically disadvantaged individuals and communities [15]. Using data from the World Health Organizations' Study on Global Aging and Adult Health (SAGE), Lloyd-Sherlock et al. reported that compared to five (5) other low- and middle-income countries the Ghanaian sample had the lowest prevalence of hypertension awareness of just 23.3% compared to a range of about 38% for India and South Africa to 72.1% for Russian Federation [16]. The increasing hypertension prevalence [4] and the serious consequences of the diseases warrant that attention be given to public education efforts as a primary prevention measure to address this growing public health problem.

In this study, the age of the respondents was found to be significantly associated with either having both elevated systolic and diastolic blood pressure or having isolated elevated systolic blood pressure. Older adults (50 years and above) had elevated isolated systolic consistent with other findings; older respondents had elevated isolated systolic blood pressure. This was consistent with similar studies in rural and urban India [17]. Contrarily to our expectation a greater proportion of respondents below 50 years had both elevated systolic and diastolic high blood pressure compared to their older counterparts. There is, however, an increase in the incidence of young adults with hypertension in other populace [18].

These findings also buttress the need for the increased call for routine checks for high blood pressure among young adults as advocated for older adults. This is because, notably, young adults with elevated systolic and diastolic pressure are likely to have a slower rate of receiving an initial diagnosis than middle-aged and older adults in their study [19]. This can easily lead to a various health complication.

Also, more than half (55%) of the respondents in this study reported that they had a family history of hypertension. This is much higher than the 35.7 % reported in a study on the frequency of hypertension and prehypertension among adults in Hohoe, a municipality in the Volta region of Ghana [20]. A review of population-based studies on hypertension in Ghana indicated that hypertension was positively associated with a positive family history of hypertension [21]. However, a study in Saudi Arabia, which examined the risk factors for diabetes and hypertension among expatriate workers, revealed that family history was not significantly associated with hypertension [22].

The predominant occupation of the respondents in this study was trading. This is likely to predispose them to hypertension due to the job-related stress and inappropriate food consumption and meal patterns. Concerning the level of income of the respondents, the majority of them were earning GHS200.00 or less. This low-income level may influence the dietary habits of the respondents and their ability to purchase foods beneficial for hypertension prevention and control [23, 24]. However, the systematic review by Addo and colleagues on hypertension in Ghana showed an inconclusive relationship between income level and hypertension [5].

Our study found significant differences in nutritional status (BMI) between participants with both elevated systolic and diastolic blood pressure and those with isolated SBP. The association between BMI and blood pressure is well established with several studies reporting higher likelihood of elevated BP among overweight and obese individuals [25–27]. A study assessing the determinants of isolated systolic hypertension in North India among both urban and rural areas showed that BMI was a significant independent predictor of isolated systolic hypertension [17]. In our study a significantly higher proportion of participants with both elevated SBP and DBP were overweight or obese compared to those with only isolated SBP. Similarly, a study conducted in South Korea found that having BMI ≥23 was associated with uncontrolled hypertension among elderly people [28]. Zhang and colleagues in their research among rural Chinese women also reported that although obese women had an increased risk of hypertension, “BMI was more related to isolated diastolic hypertension than to isolated systolic hypertension” [29].

The frequency of consuming food from various food groups was also found to be a contributory factor to having either an isolated elevated SBP or both elevated SBP and DBP. This research found out that respondents consumed fewer green leafy vegetables and fruits, which generally tend to be higher in potassium needed in the management of hypertension and had increased intake of fats and oils. A cross-sectional study on the prevalence and correlates of hypertension among rural populations in Sub-Saharan African revealed that frequent intake of fruit and vegetables was associated with lower blood pressure measures [30]. Additionally, a research in Chile showed that increased intakes of fruits and vegetables reduced the systolic blood pressure [31]. Some studies have identified lower income levels as a contributory factor to lower intake of fruits and vegetables and higher consumption of fatty foods [23, 32].

Generally, although the restriction of salted foods is considered an appropriate strategy for the control of high blood pressure adherence to this recommendation is often a challenge [33]. In the present study, the consumption of salted fish and fats and oils was higher than recommended for individuals with hypertension. It was observed that median salted fish consumption was about 3 times in the past week and while the present study did not show a significant relationship between salted fish consumption and having either both elevated SBP and DBP or isolated SBP, this link has been established in other studies. For example, among Japanese men, systolic blood pressure was significantly higher in the higher quintiles of salt intake while in India total dietary salt intake was a significant risk factor for hypertension even after controlling for potential confounders [34, 35]. Conversely, significant reduction in dietary sodium intake has been identified as an effective way of lowering SBP in Asian patients [36, 37]. Thus, measures to reduce salt intake may contribute to effective hypertension management in the study population.

Food groups such as legumes and green leafy vegetables that have potential benefits for hypertension management [38, 39] were minimally consumed by respondents in the week preceding the survey. Despite the benefits of legume consumption to hypertension management, results from this study corroborate others from even developed countries such as the United States reporting only about 8% adults consuming legumes on any given day [40].

The intake of dairy foods, which are good sources of calcium and potassium, was very low among the study participants. A systematic review and meta-analysis of elevated blood pressure and consumption of dairy foods showed that consumption of low-fat dairy foods was associated with a 13% reduction in risk of high blood pressure [41]. However, although the DASH diet recommends foods high in calcium, a study in rural African community did not find an association between the frequencies of consuming dairy products and the prevalence of hypertension [30].

The findings of this study provide useful information on dietary habits of people with hypertension in a low-income rural community which may inform hypertension management services. The study also points to the need for education and awareness among the general populace to foster better health-seeking behaviours for hypertension screening and management. Furthermore, lessons from this study will assist in developing programs for healthy ageing consistent with the World Health Organizations global strategy on ageing and health [2].

## Figures and Tables

**Table 1:** Selected background sociodemographic characteristics of study respondents.

Characteristic	n=63
**Sex**	
Female	55.6 (35)
Male	44.4 (28)
**Age in years**	54.5 ± 13.8
**Ethnicity**	
Krobo	76.2 (48)
Other	23.8 (15)
**Religious affiliation**	
Christianity	90.5 (57)
Islam and traditional religion	9.5 (6)
**Marital status**	
Married / Cohabiting	55.6 (35)
Single	44.4 (28)
**Education**	
No education	22.2 (14)
Primary	17.5 (11)
Junior High	38.1 (24)
Senior high/Technical and Vocational	4.8 (3)
Tertiary and above	17.4 (11)
**Employment status**	
Unemployed	3.2 (2)
Farming and fisher folks	17.5 (11)
Traders	38.1 (24)
Salaried workers	22.2 (14)
Artisans and labourers and others	19.0 (12)
**Household size**	5.1 ± 2.6
**Households income**	
≤ 200 GH	61.9 (39)
>200–600	25.4 (16)
>600	12.7 (8)

Values represent percentage (number) or mean ± standard deviation.

**Table 2:** Comparison of sociodemographic, anthropometric, and family health characteristics between participants with both elevated SBP^1^ and DBP^2^ and those with only elevated SBP.

Characteristic	Hypertension Classification		P-Value^3^
	Both elevated SBP and DBP (n=45)	Isolated elevated SBP (n=18)	
Age in years			0.027
26–49	46.7 (21)	16.7 (3)	
50–95	53.3 (24)	83.3 (15)	
Education			0.502
No formal	20.0 (9)	27.8 (5)	
Some formal education	80.0 (13)	72.2 (13)	
Family history of at least one chronic disease			0.935
No	37.8 (17)	38.9 (7)	
Yes	62.2 (28)	61.1 (11)	
BMI			0.047
Normal (18.5–24.9)	28.9 (13)	55.6 (10)	
Overweight/obese (≥25)	71.1 (32)	44.4 (8)	

Values represent % (n). ^1^Elevated systolic blood pressure (≥ 140 mmHg); ^2^elevated diastolic blood pressure (≥ 90mmHg); ^3^Pearson Chi-Square Statistic.

## Data Availability

Data will be made available when requested.
